# Endosymbiont Infections in Korean Insects: Patterns Across Orders and Habitat Types

**DOI:** 10.3390/insects17010071

**Published:** 2026-01-07

**Authors:** Jae-Yeon Kang, Gilsang Jeong, In Jung An, Kihyun Kim, Se-hwan Son, Soyeon Park

**Affiliations:** 1 Ecological Technology Research Team, National Institute of Ecology, 1210 Geumgang-ro, Maseo-myeon, Seocheon-gun 33657, Chungcheongnam-do, Republic of Korea; jaeyoni@nie.re.kr; 2 Division of Restoration Research, Research Center for Endangered Species, National Institute of Ecology, 23 Gowol-gil, Yeongyang-eup, Yeongyang-gun 36531, Gyeongsangbuk-do, Republic of Korea; gilsangj@nie.re.kr; 3 Invasive Alien Species Team, National Institute of Ecology, 1210 Geumgang-ro, Maseo-myeon, Seocheon-gun 33657, Chungcheongnam-do, Republic of Korea; injungg0917@nie.re.kr; 4 Restoration Ecology Research Team, National Institute of Ecology, 1210 Geumgang-ro, Maseo-myeon, Seocheon-gun 33657, Chungcheongnam-do, Republic of Korea; k717kh@nie.re.kr; 5 SH Aquatic Biome Research, 257-20 Munwha-ro, Asan-si 31523, Chungcheongnam-do, Republic of Korea; son-s.h@daum.net; 6 Interdisciplinary Program of EcoCreative, Ewha Womans University, 52 Ewhayeodae-gil, Seodaemun-gu, Seoul 03760, Republic of Korea

**Keywords:** endosymbiont infection patterns, co-infection, *Wolbachia*, habitat type, taxonomic variation, Korean insects

## Abstract

Endosymbiotic bacteria influence the ecology and evolution of insects through complex associations within host cells. To explore how these relationships vary among environments and taxa, we examined 1028 insect specimens from 14 orders across Korea for infections by three representative endosymbionts (*Wolbachia*, *Rickettsia*, and *Spiroplasma*). Overall, 33.8% of specimens were infected, with single infections predominating and co-infections remaining relatively less common. Weak-to-modest but statistically significant associations were detected between several symbiont pairs (*Rickettsia–Spiroplasma*, *Wolbachia–Spiroplasma*, and *Wolbachia–Rickettsia*). Infection rates exhibited no significant variation among host orders except for *Spiroplasma*, and *Wolbachia* infections were more frequently detected in terrestrial than in aquatic insects. These results indicate that endosymbiont infection patterns might be shaped by factors operating at multiple biological scales, including host taxonomy and habitat types. As this study relied on polymerase chain reaction detection, infection frequencies should be interpreted as comparative rather than absolute measures. This survey provides baseline data that might help characterize regional patterns of endosymbiont distributions and their variation across taxonomic and ecological contexts.

## Simple Summary

Endosymbiotic bacteria—microbes that live inside insect cells—play important roles in shaping the biology and evolution of their hosts. In this study, we examined more than 1000 insects from 14 different orders across Korea to explore how three representative endosymbionts (*Wolbachia*, *Rickettsia*, and *Spiroplasma*) are distributed among hosts living in different environments. Single infections predominated, while co-infections were infrequent among the infected insects. Overall associations among symbiont pairs were weak, but varied among insect orders, with significant associations concentrated in Coleoptera and Hemiptera. Infection rates were broadly similar across most host orders, although *Spiroplasma* displayed detectable order-level differences. In addition, *Wolbachia* infections were more frequently detected in terrestrial than in aquatic insects. These findings indicate that endosymbiont infection patterns might be shaped by multiple factors operating at different biological scales. This study provides baseline data on endosymbiont distributions in Korean insects, offering additional context for understanding regional variation in these host–microbe associations.

## 1. Introduction

Organisms exist not as isolated entities but as a holobiont that comprises a host as well as its associated microbiota [1]. These microbial consortia exert broad influences on host physiology, ecology, and evolution [2,3]. Among these associations, intracellular endosymbionts represent the most intimate form of symbiosis, directly shaping host reproductive systems and patterns of genetic inheritance [4]. Representative endosymbionts include *Wolbachia*, *Rickettsia*, and *Spiroplasma*, with *Wolbachia* particularly well known for manipulating host reproduction through cytoplasmic incompatibility, parthenogenesis induction, male killing, and feminization, thereby enhancing maternal transmission [5,6]. Such reproductive alterations can influence host sex ratios and have been proposed as contributors to evolutionary processes such as speciation [5].

Due to these striking reproductive effects, endosymbionts have attracted broad attention across arthropods, especially insects, where numerous surveys have been conducted. *Wolbachia* is estimated to infect roughly 40–60% of arthropod species [7,8] and has become the most intensively studied endosymbiont owing to its applications in biological control and suppression of vector-borne diseases [9,10]. In contrast, broad-scale comparative surveys that simultaneously screen multiple reproductive manipulators, including *Rickettsia* and *Spiroplasma*, remain relatively limited [5]. Many previous investigations have been motivated by applied objectives, concentrating on particular insect groups (e.g., pest or vector species) or on *Wolbachia* alone [11]. Similar tendencies appear in Korea, where endosymbiont screening has largely been confined to a few taxa such as ants, beetles, or mosquitoes, generally targeting *Wolbachia* or other single symbionts [12,13,14]. To date, little coordinated effort has been directed toward characterizing the distribution and co-occurrence (co-infection) patterns of multiple endosymbionts across diverse insect orders or ecological contexts in Korea.

Multiple factors are likely to shape the observed infection patterns. Most endosymbionts rely primarily on vertical (maternal) transmission via the host’s egg cytoplasm [15], although horizontal transmission across phylogenetically distant hosts has also been documented for several symbionts including *Wolbachia* [16,17] and *Spiroplasma* [18]. The establishment and maintenance of endosymbiont infections likely involve factors operating at different biological scales, ranging from ecological contexts to host taxonomic affiliations, suggesting that infection patterns observed in nature emerge from multilevel processes. Yet, most prior studies have focused on specific mechanisms or laboratory models, rarely integrating ecological or habitat-level variables into broad-scale analyses [8,19].

In this context, comparative analyses that span broad taxonomic and ecological diversity can provide important insight into the eco-evolutionary processes underlying endosymbiont transmission and establishment. The Korean insect fauna, which encompasses more than 20 orders and a wide range of habitat types, therefore presents an effective setting for exploring ecological and phylogenetic factors influencing endosymbiont infections. The presence of distinct habitat guilds within a shared geographic region offers a direct opportunity to examine how environmental conditions shape symbiont persistence and transmission dynamics.

In this study, we screened three major reproductive endosymbionts, i.e., *Wolbachia*, *Rickettsia*, and *Spiroplasma*, across insect specimens collected throughout Korea. Infection and co-infection patterns were compared across insect orders and across habitat types (aquatic and terrestrial). Our specific goals were to (1) characterize taxon-specific infection profiles of each symbiont, (2) evaluate co-occurrence and potential associations among symbionts, and (3) examine ecological differentiation in infection prevalence between aquatic and terrestrial insects. However, instead of testing specific mechanistic hypotheses, this study aims to provide a comprehensive baseline dataset that highlights potential multilevel ecological and evolutionary drivers of endosymbiont infection patterns and establishes a foundation for future hypothesis-driven research.

## 2. Materials and Methods

### 2.1. Sample Collection and Extraction of Genomic DNA

Insects inhabiting Korea were collected randomly throughout the year, regardless of region. The collected specimens were initially identified to the species or genus level based on their morphological characteristics; species and family names followed the National List of Species of Korea [20]. In this study, we collected a total of 1028 individuals representing approximately 230 species (with an additional 19 taxa identified only to the genus level) across 14 insect orders. The original infection records used in this analysis were derived from the National Institute of Ecology’s 2020 annual report, which compiled survey data collected between 2017 and 2020 [21]. Species identification results and detailed sampling information are provided in the Supplementary Materials (Table S1). Specimens were classified as aquatic or terrestrial based on the primary habitat of their immature stages, regardless of the life stage at collection. After collection and morphological identification, specimens were preserved in 100% ethanol and stored at −20 °C until genomic DNA extraction. For DNA extraction, the insect exoskeleton was thoroughly crushed with a pestle. Smaller specimens were processed whole, whereas legs or other body parts were used for larger specimens. DNA was then extracted in accordance with the manufacturer’s protocol for the DNeasy Blood & Tissue Kit (Qiagen, Hilden, Germany).

### 2.2. Host Identification

To complement this morphology-based identification at the genetic level, the mitochondrial cytochrome oxidase I (COI) gene region of approximately 650 bp was amplified from 727 individuals out of the 1028 total specimens. COI analysis served as a supplementary step to verify the initial morphological identification and to confirm species identity. For amplification, the universal primer pair LCO1490–HCO2198 [22] was primarily used, applying polymerase chain reaction (PCR) conditions of 95 °C for 3 min; 35 cycles of 94 °C for 1 min, 48 °C for 1 min, and 72 °C for 1 min; followed by 72 °C for 5 min. For samples that did not amplify successfully, the dgLCO–dgHCO primer pair [23] was used under revised conditions of 95 °C for 3 min; 35 cycles of 94 °C for 1 min, 50 °C for 1 min, and 72 °C for 1 min; followed by 72 °C for 5 min [23,24]. Sequencing yielded fragments of roughly 618 bp, which were used to construct interspecific phylogenetic trees for internal verification but were not incorporated into this paper. The obtained sequences were edited using MEGA X (version 10.2.6) [25], and molecular species identification was carried out by comparison with the GenBank database using Basic Local Alignment Search Tool (BLAST) during the course of the study for internal species identification.

### 2.3. Endosymbiont Detection and Validation

Infection status was confirmed for three endosymbionts (i.e., *Wolbachia*, *Spiroplasma*, and *Rickettsia*). PCR was performed using specific primers for each endosymbiont [14,26], and infection was detected based on PCR amplification results. Each PCR run included a negative control (nuclease-free water) and a positive control (a previously validated PCR-positive sample for each target endosymbiont). The PCR conditions for *Wolbachia* were 94 °C for 2 min; 38 cycles of 94 °C for 30 s, 55 °C for 45 s, and 72 °C for 90 s; followed by a final extension at 72 °C for 10 min. For *Spiroplasma* and *Rickettsia*, PCR conditions were 94 °C for 3 min; 35 cycles of 94 °C for 1 min, 60 °C for 1 min, and 72 °C for 1 min; followed by 72 °C for 5 min. Since the purpose of this study was to confirm infection status, we did not perform further sequencing of PCR products. Primer information used for all PCR is provided in Table S2.

To verify detection accuracy, a subset of PCR-positive samples representing diverse insect orders was subjected to Sanger sequencing. A total of 55 sequences were obtained: 24 *Wolbachia* sequences amplified using *ftsZ* gene primers [27], which is commonly used for multi locus sequence typing (MLST) of *Wolbachia*; 22 *Spiroplasma* 16S rDNA sequences; and 9 *Rickettsia* 16S rDNA sequences. PCR conditions for *ftsZ* amplification were 94 °C for 2 min; 35 cycles of 94 °C for 30 s, 54 °C for 45 s, and 72 °C for 1 min; followed by 72 °C for 10 min. Sequence identity was confirmed by BLAST (https://blast.ncbi.nlm.nih.gov/Blast.cgi, accessed on 22 December 2025) analysis against the National Center for Biotechnology Information (NCBI) GenBank database. All sequences showed ≥97.7% identity to their respective target genera, confirming the specificity of the detection primers. Validated sequences have been deposited in GenBank, and detailed validation results including accession numbers are provided in Table S3.

### 2.4. Statistical Analyses

We quantitatively examined infection patterns of three sex-manipulating endosymbiotic bacteria, i.e., *Spiroplasma*, *Rickettsia*, and *Wolbachia*, across 1028 insect specimens representing 14 orders. To do so, pairwise associations among symbiont infections were evaluated using a Phi coefficient (φ) to measure co-occurrence strength. Subsequently, Chi-square tests (χ^2^) were used to assess statistical independence among infection pairs. Phi coefficients range from −1 to +1, with values near 0 indicating independence.

Differences in infection prevalence among insect orders were tested using Kruskal–Wallis tests. For each symbiont, we included only orders with detectable infections to assess infection intensity among susceptible host lineages. When significant differences were found, we further performed Dunn’s post hoc multiple comparisons using Holm-adjusted *p*-values (<0.05).

Next, we evaluated differences in infection rates between ecological traits (e.g., aquatic and terrestrial) using the Mann–Whitney U test with Holm correction. Unlike the order-level analysis, this comparison included all sampled orders to evaluate ecological effects on endosymbiont establishment.

All statistical analyses were performed in Python 3.12.4 using a Jupyter Notebook (version 7.0.8) within the Anaconda environment. We used the following packages: scipy.stats (version 1.16.3 for Kruskal–Wallis, Mann–Whitney U, and Chi-square tests), scikit-posthocs (version 0.11.4 for Dunn’s post hoc comparisons), pandas (version 2.3.3) and NumPy (version 1.26.4 for data manipulation), and Matplotlib (version 3.10.6 for visualization). Code development was assisted by ChatGPT 5.0 (OpenAI).

## 3. Results

### 3.1. Infection Prevalence Across Insect Orders

**Figure 1:**
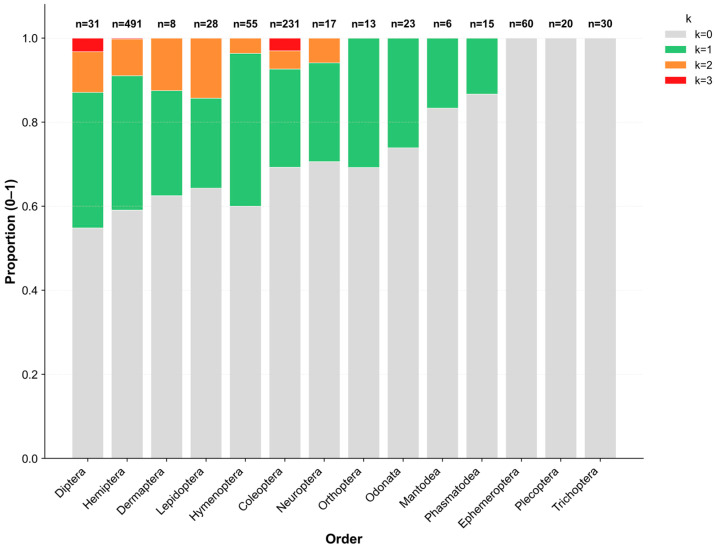
Proportional distribution of co-infection counts (k = 0–3) across 14 insect orders. Bars represent the proportion of individuals within each order carrying 0, 1, 2, or 3 sex-manipulating endosymbiont species, respectively. Color intensity indicates the number of co-infected symbionts. The numbers above the bars show the number of individuals analyzed per order (n).

A total of 1028 insect specimens representing 14 taxonomic orders were screened for infections by three endosymbiotic bacteria, e.g., *Spiroplasma*, *Wolbachia*, and *Rickettsia*. Among these bacteria, 347 individuals (33.8%) carried at least one endosymbiont, whereas 681 individuals (66.2%) showed no detectable infections. Single infections predominated, while co-infections were comparatively uncommon: 70 individuals (6.8%) harbored two symbionts, and 11 individuals (1.1%) harbored three symbionts simultaneously (Figure 1).

Infection prevalence varied noticeably among insect orders, although the sample sizes differed considerably (range: 6–491 individuals per order). Diptera and Hemiptera exhibited the highest infection rates (45.2% and 42.6%, respectively), with Diptera showing the largest proportion of co-infections (12.9%). Conversely, several other orders showed comparatively low infection frequencies, largely dominated by single infections. Notably, among aquatic insects, all 110 individuals of the EPT orders (i.e., Ephemeroptera, Plecoptera, and Trichoptera) were uninfected. In contrast, Odonata species displayed a moderate infection prevalence of 26.1%.

### 3.2. Patterns of Symbiont Co-Infection

**Figure 2:**
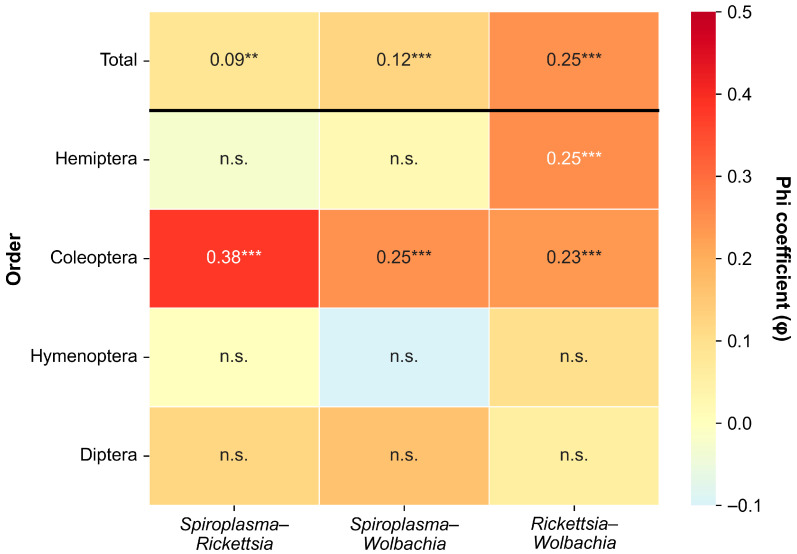
Phi coefficient (φ) matrix showing pairwise co-infection associations among three endosymbionts (*Spiroplasma*, *Rickettsia*, and *Wolbachia*) for total samples and by insect order. Only orders with n ≥ 30 and at least one detected infection are shown. ** *p* < 0.01, *** *p* < 0.001, n.s. = not significant.

The associations among the three endosymbionts were weak but statistically significant (Figure 2, Table 1). The *Rickettsia–Wolbachia* combination showed the highest association (φ = 0.25, χ^2^ = 62.35, *p* < 0.001), followed by *Spiroplasma–Wolbachia* (φ = 0.12, χ^2^ = 15.37, *p* < 0.001) and *Spiroplasma–Rickettsia* (φ = 0.09, χ^2^ = 7.67, *p* < 0.01).

However, at the order level, co-infection patterns varied among insect orders. In Coleoptera, the *Spiroplasma–Rickettsia* association was moderate (φ = 0.38, χ^2^ = 33.56, *p* < 0.001). Coleoptera showed significant positive associations across all three symbiont pairs (all *p* < 0.001). Hemiptera showed a different pattern, with only the *Rickettsia*–*Wolbachia* combination showing a significant association (φ = 0.25, *p* < 0.001); *Spiroplasma*-related pairs were not significant. Diptera and Hymenoptera showed no significant associations for any symbiont pair (all *p* > 0.05).

### 3.3. Prevalence of Individual Symbiont Infection Among Insect Orders

**Figure 3:**
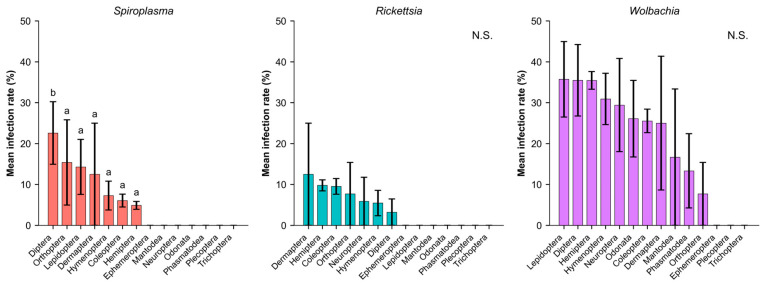
Infection prevalence of three endosymbionts across insect orders. Different letters indicate significant differences based on Holm-corrected Dunn’s post hoc tests (*p* < 0.05). N.S. denotes cases with no significant differences among orders. Bars represent mean ± SE. Statistical comparisons for each symbiont include only the orders in which that symbiont was detected.

Infection prevalence showed diverse patterns across insect orders and endosymbionts (Figure 3). Since each symbiont displayed distinct host range patterns, with many orders showing no detectable infections for individual symbionts, statistical comparisons were conducted separately for each symbiont and limited to the orders in which infections were detected. *Spiroplasma* exhibited significant differences among the seven orders where it occurred, and Holm-corrected Dunn’s post hoc tests indicated that Diptera (22.6%) had significantly higher infection rates than most other infected orders. Within these seven orders, Diptera formed a distinct group, whereas the remaining six orders showed broadly similar infection levels (6.1–15.4%). *Wolbachia* was detected in 11 orders, with infection rates ranging from 7.7% to 35.7%; however, no significant differences were identified among the orders where it occurred (H = 14.63, *p* = 0.146).

### 3.4. Differences in Symbiont Infection Prevalence Between Aquatic and Terrestrial Insects

Next, to examine ecological differentiation in infection prevalence between aquatic and terrestrial insects, we compared endosymbiont infection rates between the two habitat types. Mann–Whitney U tests with Holm correction revealed significant differences for all three symbionts (Table 2).

**Figure 4:**
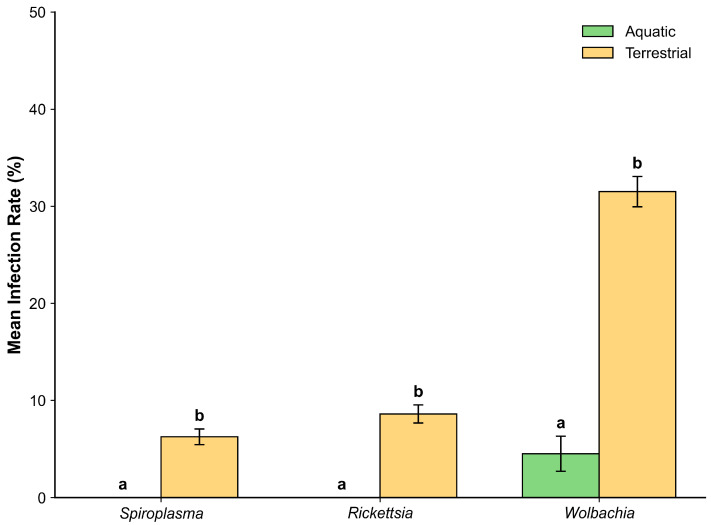
Infection rates of three endosymbionts in aquatic (green) and terrestrial (yellow) insects. Different letters indicate significant differences based on Holm-corrected Mann–Whitney U tests (*p* < 0.05). Bars represent mean ± SE.

*Wolbachia* showed the strongest pattern (U = 43,449.5, *p* < 0.001), with terrestrial insects (31.5% ± 1.6%) exhibiting markedly higher infection rates than aquatic insects (4.5 ± 1.8%). *Rickettsia* (U = 54,397, *p* < 0.01) and *Spiroplasma* (U = 55,793, *p* < 0.01) were also significantly more frequent in terrestrial insects (8.6 ± 0.9% and 6.3 ± 0.8%, respectively), while being entirely absent in aquatic insects (0%). Taken together, these results quantitatively reinforce the complete absence of endosymbionts observed in the EPT orders (Figure 4).

## 4. Discussion

This study investigated the infection status of three representative endosymbionts (*Wolbachia*, *Rickettsia*, *Spiroplasma*) across 14 insect orders in Korea and compared infection patterns according to taxonomic groups and habitat types. Infections were mainly observed as single infections, while co-infections involving two or more endosymbionts appeared relatively limited. Pairwise analyses revealed weak but significant positive associations among *Wolbachia*, *Rickettsia*, and *Spiroplasma*. Among host orders, only *Spiroplasma* exhibited significant variation in infection rates. Distinct differences in infection rates were observed between aquatic and terrestrial habitats, particularly for *Wolbachia*. These patterns suggest that endosymbiont infections might be shaped by factors operating at multiple biological scales.

Co-infections were relatively uncommon, with most infected insects harboring only a single symbiont. Among the observed co-infections, weak-to-modest positive associations were detected for *Rickettsia*–*Spiroplasma*, *Wolbachia*–*Spiroplasma*, and *Wolbachia*–*Rickettsia* pairs. Similar co-infection patterns among these symbionts have been reported only occasionally in arthropods, including beetles, mites, and aphids [28,29,30]. Overall, these findings suggest that detectable co-infection occurs mainly among a few symbiont pairs and remains infrequent across natural insect taxa.

Weinert et al. [7] reported significant order-level differences in infection prevalence for several endosymbionts, although their analysis did not include *Spiroplasma*. Conversely, *Wolbachia* and *Rickettsia* displayed no significant order-level differences across the 14 sampled insect orders in this study, whereas only *Spiroplasma* exhibited detectable variation. This contrast might reflect differences in geographic scope, sampling coverage, and taxonomic representation between global meta-analyses and region-focused surveys. *Spiroplasma’s* order-specific variation is notable given that this symbiont was not included in previous large-scale comparative studies, although strain-level analysis was beyond the scope of this study. Overall, these findings suggest that regional datasets can complement global summaries by providing additional context on variations in endosymbiont distributions across taxonomic groups and spatial scales.

Notable differences in infection patterns were observed between terrestrial and aquatic habitat types, with terrestrial insects exhibiting higher infection frequencies, particularly for *Wolbachia*. In our dataset, the specimen-level prevalence of *Wolbachia* in aquatic insects (Odonata + EPT) was 4.5%. This value is not directly comparable to the species-level incidence of approximately 52% estimated by Sazama et al. [31], as the two studies used different analytical units (specimens vs. species). Notably, none of the three endosymbionts was detected in any of the 110 EPT specimens examined, and the observed aquatic *Wolbachia* infections were restricted to Odonata. This EPT-specific absence is broadly consistent with the findings of Ayayee et al. [32], who similarly detected no *Wolbachia* in Ephemeroptera using PCR-based screening targeting *wsp*, although low-level signals were suggested in their microbiome-based analyses. Sazama et al. [31] likewise highlighted persistent uncertainty for EPT orders due to limited sampling and low infection rates. The discrepancy between our low aquatic infection rate and higher values reported in other studies likely reflects differences in taxonomic coverage: our aquatic sampling included only Odonata and EPT, whereas studies reporting higher prevalence often included aquatic Diptera, Coleoptera, or Hemiptera. The consistent absence or minimal detection of endosymbionts in EPT across multiple studies, now reinforced by our examination of 110 EPT specimens, suggests that these orders might face constraints that limit symbiont establishment. Collectively, these findings underscore that habitat type and host order jointly influence endosymbiont distribution patterns.

Several methodological limitations should be noted. Sampling was uneven across insect orders, which might have reduced statistical power for detecting low-prevalence infections or subtle taxonomic differences. Standard PCR-based screening can also underestimate low-titer infections or symbionts with divergent sequences, a limitation noted in previous studies of heritable bacterial symbionts [5,30]. DNA extraction from legs or other body parts for larger specimens may have reduced detection sensitivity, as some endosymbionts exhibit tissue-specific distributions. While leg-derived DNA has been shown to be sufficient for detecting *Wolbachia* [33], detection efficiency for other endosymbionts such as *Spiroplasma* and *Rickettsia* may be more limited. In addition, the cross-sectional nature of this survey precluded temporal or seasonal inference regarding infection dynamics. Consequently, the patterns presented in this study should be viewed as comparative indicators rather than precise estimates of true infection prevalence.

## 5. Conclusions

Our findings indicate that endosymbiont infection patterns in Korean insects might be shaped by multiple interacting factors, as suggested by the relative infrequency of co-infections among symbionts, the presence of order-level variation only in *Spiroplasma*, and the notable differences associated with habitat types. These multilevel patterns may suggest that no single determinant alone can fully explain the distribution of infections across taxa and environments and that both taxonomic and ecological contexts merit consideration when interpreting broad-scale symbiont prevalence structures.

Despite methodological limitations discussed above, this study provides several important contributions. First, it increased the relatively small number of multi-order regional surveys in East Asia that simultaneously screened three major endosymbionts, thereby expanding geographic representation in global endosymbiont datasets. Second, it included aquatic insect orders (Ephemeroptera, Plecoptera, and Trichoptera) that have been historically underrepresented in prevalence surveys, offering new baseline data for groups noted as difficult to sample in previous large-scale work [31]. Third, it incorporated *Spiroplasma* into a multi-symbiont framework, adding comparative data that were absent from earlier global summaries such as Weinert et al. [7]. Together, these dataset-level contributions have broadened the empirical basis for understanding regional variation in endosymbiont communities and provided opportunities for generating hypotheses about the relative roles of ecological and taxonomic factors in shaping infection patterns. Future research that integrates quantitative assays, strain-level characterization, and expanded temporal and spatial sampling across Asian ecosystems might help further clarify the processes underlying these multilevel patterns.

## Figures and Tables

**Table 1:** Pairwise associations among endosymbionts based on Chi-square tests. φ indicates the strength of association; −log_10_(p) indicates statistical significance.

Order	n	Pair	φ	χ^2^	−log_10_(p)	*p*-Value
Total	1028	*Spiroplasma*–*Rickettsia*	0.09	7.67	2.3	<0.01
		*Spiroplasma*–*Wolbachia*	0.12	15.37	4.1	<0.001
		*Rickettsia*–*Wolbachia*	0.25	62.35	14.5	<0.001
Coleoptera	231	*Spiroplasma*–*Rickettsia*	0.38	33.56	8.2	<0.001
		*Spiroplasma*–*Wolbachia*	0.25	14.41	3.8	<0.001
		*Rickettsia*–*Wolbachia*	0.23	12.22	3.3	<0.001
Hemiptera	491	*Spiroplasma*–*Rickettsia*	—	—	—	n.s.
		*Spiroplasma*–*Wolbachia*	—	—	—	n.s.
		*Rickettsia*–*Wolbachia*	0.25	30.69	7.5	<0.001
Diptera	42	*Spiroplasma*–*Rickettsia*	—	—	—	n.s.
		*Spiroplasma*–*Wolbachia*	—	—	—	n.s.
		*Rickettsia*–*Wolbachia*	—	—	—	n.s.
Hymenoptera	31	*Spiroplasma*–*Rickettsia*	—	—	—	n.s.
		*Spiroplasma*–*Wolbachia*	—	—	—	n.s.
		*Rickettsia*–*Wolbachia*	—	—	—	n.s.

**Table 2:** Comparison of infection prevalence for three endosymbionts (e.g., *Spiroplasma*, *Rickettsia*, and *Wolbachia*) between aquatic and terrestrial insect groups (Mann–Whitney U test, *p* < 0.05).

Symbiont	Aquatic (Mean ± SE, %)	Terrestrial (Mean ± SE, %)	U_Stat	*p*-Value
*Spiroplasma*	-	6.26 ± 0.81	55,793.5	<0.01
*Rickettsia*	-	8.60 ± 0.94	54,397.0	<0.001
*Wolbachia*	4.51 ± 1.81	31.51 ± 1.55	43,449.5	<0.001

## Data Availability

The species identification results and detailed sampling information supporting the reported results are available in the Supplementary Materials, which are publicly accessible at [http://doi.or.kr/10.22756/GEO.20250000000992] (accessed on 26 December 2025).

## Supplementary Materials

The following supporting information can be downloaded at: https://www.mdpi.com/article/10.3390/insects17010071/s1. Table S1: Sample information for all 1028 insect specimens, including taxonomic identification, collection data, habitat classification, life stage, and infection status. Table S2: Primer sets used for PCR amplification of three endosymbionts (*Wolbachia*, *Spiroplasma*, and *Rickettsia*) and host COI gene, including target loci, primer sequences, expected amplicon sizes, and references. Table S3: Sequence validation results for 55 PCR-positive samples, including BLAST identity scores, query coverage, and GenBank accession numbers.

## Abbreviations

COI	Cytochrome oxidase I
EPT	Ephemeroptera, Plecoptera, and Trichoptera
PCR	Polymerase chain reaction
MLST	multilocus sequence typing
BLAST	Basic Local Alignment Search Tool
NCBI	National Center for Biotechnology Information
