# Correction: Kang et al. Endosymbiont Infections in Korean Insects: Patterns Across Orders and Habitat Types. *Insects* 2026, *17*, 71

**DOI:** 10.3390/insects17050498

**Published:** 2026-05-14

**Authors:** Jae-Yeon Kang, Gilsang Jeong, In Jung An, Kihyun Kim, Se-hwan Son, Soyeon Park

**Affiliations:** 1 Ecological Technology Research Team, National Institute of Ecology, 1210 Geumgang-ro, Maseo-myeon, Seocheon-gun 33657, Chungcheongnam-do, Republic of Korea; jaeyoni@nie.re.kr; 2 Division of Restoration Research, Research Center for Endangered Species, National Institute of Ecology, 23 Gowol-gil, Yeongyang-eup, Yeongyang-gun 36531, Gyeongsangbuk-do, Republic of Korea; 3 Invasive Alien Species Team, National Institute of Ecology, 1210 Geumgang-ro, Maseo-myeon, Seocheon-gun 33657, Chungcheongnam-do, Republic of Korea; 4 Restoration Ecology Research Team, National Institute of Ecology, 1210 Geumgang-ro, Maseo-myeon, Seocheon-gun 33657, Chungcheongnam-do, Republic of Korea; 5 SH Aquatic Biome Research, 257-20 Munwha-ro, Asan-si 31523, Chungcheongnam-do, Republic of Korea; 6 Interdisciplinary Program of EcoCreative, Ewha Womans University, 52 Ewhayeodae-gil, Seodaemun-gu, Seoul 03760, Republic of Korea

**Additional Affiliation(s)**

In the published publication [1], there was an error regarding the affiliation for Soyeon Park. In addition to affiliation 1, the updated affiliation should include the following: 6. Interdisciplinary Program of EcoCreative, Ewha Womans University, 52 Ewhayeodae-gil, Seodaemun-gu, Seoul 03760, Republic of Korea.

The authors state that the scientific conclusions are unaffected. This correction was approved by the Academic Editor. The original publication has also been updated.
